# Genomic predictions of genetic variances and correlations among traits for breeding crosses in soybean

**DOI:** 10.1038/s41437-024-00703-3

**Published:** 2024-07-12

**Authors:** Cleiton A. Wartha, Aaron J. Lorenz

**Affiliations:** Department of Agronomy and Plant Genetics, University of Minnesota, St. Paul, MN, USA

**Keywords:** Plant breeding, Plant hybridization

## Abstract

Parental selection is perhaps the most critical decision a breeder makes, establishing the foundation of the entire program for years to come. Cross selection based on predicted mean and genetic variance can be further expanded to multiple-trait improvement by predicting the genetic correlation (${r}_{G}$) between pairs of traits. Our objective was to empirically assess the ability to predict the family mean, genetic variance, superior progeny mean and genetic correlation through genomic prediction in a soybean population. Data made available through the Soybean Nested Association Mapping project included phenotypic data on seven traits (days to maturity, lodging, oil, plant height, protein, seed size, and seed yield) for 39 families. Training population composition followed a leave-one-family-out cross-validation scheme, with the validation family genetic parameters predicted using the remaining families as the training set. The predictive abilities for family mean and superior progeny mean were significant for all traits while predictive ability of genetic variance was significant for four traits. We were able to validate significant predictive abilities of ${r}_{G}$ for 18 out of 21 (86%) pairwise trait combinations (*P* < 0.05). The findings from this study support the use of genome-wide marker effects for predicting ${r}_{G}$ in soybean biparental crosses. If successfully implemented in breeding programs, this methodology could help to increase the rate of genetic gain for multiple correlated traits.

## Introduction

The cornerstone of plant breeding is transgressive segregation. Plant breeders strive to identify parents of superior breeding value and then intermate these parents with the goal of creating new progenies that exceed the performance of the best parent. Given the huge number of potential crosses that could be made among all suitable candidate parental lines, even within modestly sized breeding programs, methodologies that can predict the value of different parental combinations would be useful. The predicted mean and genetic variance of each possible family (wherein “family” in this context refers to those progenies that share parents and are produced from a single biparental breeding cross) could be used to discriminate among potential crosses based on an upper fraction of its progeny. This has been quantified as the *usefulness criterion* (${U}_{p}=\,\mu +\,{k}_{p}h{\sigma }_{G}$) (Schnell and Utz 1975) or the *superior progeny mean* (${\mu }_{{SP}}=\,\mu +\,{k}_{p}{\sigma }_{G}$) (Zhong and Jannink 2007), where $\mu$ is the expected family mean resulting from the cross, ${k}_{p}$ is the standardized selection differential for the proportion (*p*) of candidates selected, *h* is the selection accuracy, and ${\sigma }_{G}$ is the genetic standard deviation. Several approaches towards predicting these genetic parameters have been described in the literature using metrics such as the coefficient of parentage (Souza and Sorrells 1991), molecular marker-based genetic distance estimates (Bohn et al. 1999), and phenotype-based genetic distance estimates (Souza and Sorrells 1991; Utz et al. 2001). Limited and inconsistent outcomes in predicting the genetic variance were reported because of large standard errors, lack of trait-specific estimates, and late assessment in the breeding process when using phenotypic measurements.

The term “genomic mating” coined by Akdemir and Sánchez (2016) typically refers to approaches leveraging genome-wide molecular markers for the identification of parental combinations to maximize the likelihood of obtaining superior progenies. The genetic variance within a family is a function of the number of loci segregating, which is expected to be proportional to the coefficient of co-ancestry between the parents (Wright 1922; Souza and Sorrells 1991). However, because of random Mendelian segregation, it is also possible to identify pairs of parents that are equally related to each other yet differ in their number of polymorphisms at quantitative trait loci (QTL). In addition, alleles inherited in coupling produce a positive covariance and thus increase genetic variance, while alleles inherited in repulsion phase produce a negative covariance and thus reduce the overall genetic variance in the population (Bohn et al. 1999). Therefore, the genetic variance stems from random sampling and recombination of parental chromosomes during meiosis and could be predicted from accurate estimates of marker effects and recombination rates among the markers. Such formulas explicitly accounting for estimated marker effects and recombination rate between polymorphic markers among parents have been published (Zhong and Jannink 2007; Osthushenrich et al. 2017).

The foregoing discussion focuses on breeding for a single trait, but multi-trait improvement may be challenged by unfavorable correlations between traits due to linkage or pleiotropy (Falconer and Mackay 2009). Examples of undesirable correlations between traits in crops include seed yield and protein in soybeans (Hartwig and Hinson 1972; Wehrmann et al. 1987; Wilcox and Shibles 2001), grain yield and plant height in maize (Chi et al. 1969), and Fusarium head blight (FHB) severity and heading date in barley (Mesfin et al. 2003; Nduulu et al. 2007) and wheat (Schmolke et al. 2005). For instance, breeding and selection in soybean primarily for seed yield between 1923 and 2008 in maturity groups II-III reduced protein content by 1.9% (Rincker et al. 2014). It would therefore be desirable to select breeding populations with more favorable genetic correlations among traits to improve the likelihood of identifying progeny with favorable trait combinations.

Formulae for the genetic variance of a new population described above (Zhong and Jannink 2007; Osthushenrich et al. 2017) were expanded to calculate the expected genetic covariance between two traits in bi- or multi-parental crosses (Bonk et al. 2016; Allier et al. 2019; Neyhart et al. 2019). The correlated progeny mean on the second trait is estimated as ${\mu }_{{SP}(2)}^{C}=\,{\mu }_{(2)}+\,{k}_{p}{r}_{G(\mathrm{1,2})}{\sigma }_{G(2)}$, where ${r}_{G(\mathrm{1,2})}$ is the genetic correlation between primary (1) and secondary (2) traits. Simulating a recurrent selection program in barley, Neyhart et al. (2019) found that genetic gain for a multi-trait index was improved by 11–27% when crosses were chosen based on predicted genetic correlations as compared to random pairings of parents. Neyhart et al. (2019) suggested that in practice cross selection could be based primarily on predicted family mean given the high predictive abilities for this parameter, followed by secondary criteria of predicted genetic variance and correlation to further discriminate among all potential crosses.

The creation of novel methods for parental selection and cross design through theory and computer simulations is essential, but the utility of these methods must also be evaluated using real data to assess their potential for practical application. Varying levels of predictive abilities and usefulness of genome-wide markers to predict genetic parameters related to cross design have been reported in several previous empirical validation studies in crops such as barley (Tiede et al. 2015; Osthushenrich et al. 2018; Abed and Belzile 2019; Neyhart and Smith 2019; Neyhart et al. 2019), cassava (Wolfe et al. 2021), and maize (Lian et al. 2015; Adeyemo and Bernardo 2019). In soybeans, Jean et al. (2021) assessed success of predicting the value of a cross through a retrospective analysis of family persistence in a breeding pipeline. They found that 20 of 22 superior crosses retained by breeders were indeed predicted to have maturity-adjusted yield values above the mean, while 96.2% of all crosses predicted to have below average yield were eliminated in the breeding pipeline before advanced testing. Miller et al. (2023), using data from 42 previously made crosses, examined predictive ability of the usefulness criterion and also concluded genomic prediction of cross value holds promise to increase breeding efficiency.

Use of genomic prediction to help choose breeding crosses based on predicted family mean and variance has been empirically studied in several crops as noted above. However, limited research in this area has been reported in soybean, and limited studies on the validation of genomic predictions of genetic correlations have been reported among all crops. An exception is Neyhart et al. (2019) who examined a very limited number of traits and crosses. A main reason for this lack of empirical validation in genomic prediction of breeding cross parameters is the fact that generating the appropriate datasets of adequate size and quality is very expensive and labor intensive. It would be ideal to have many families ( > 30) with each family consisting of many progenies ( > 100) for accurate estimation of variances and correlations. Publicly available data with large population sizes and extensive reliable phenotypic data on several agronomic and seed composition traits such as the Soybean Nested Association Mapping (SoyNAM) population is a convenient resource for an initial empirical validation study in soybean. We thus present an empirical validation study of the accuracy for predicting parental selection criteria in soybean. We conducted a leave-one-family-out cross-validation scheme to evaluate our ability to predict the family mean, genetic variance, superior progeny mean and genetic correlation for seven soybean traits with deterministic equations and genome-wide marker effects estimated from a soybean nested association mapping population. We hypothesize that genomic prediction models can identify parental combinations more likely to produce new breeding families with desired means, variances, and genetic correlations.

## Material and methods

### Plant materials and phenotypic data

Publicly available genotype and phenotype data generated by the Soybean Nested Association Mapping (SoyNAM) project were used in this study (Song et al. 2017; Diers et al. 2018). The SoyNAM population consists of 40 bi-parental families created by crossing 40 founder lines to the hub parent IA3023. The composition of the founder lines was as follows: 17 elite adapted varieties and breeding lines from eight breeding programs; 15 breeding lines selected for yield and diversity from Dr. Randall Nelson’s (USDA-ARS) diversity breeding program; and 8 plant introductions (PI) with high yield in drought conditions identified by Professor James E. Specht (Supplementary Table S1) (Song et al. 2017). Each bi-parental population originally consisted of 140 F_5_-derived recombinant inbred lines (RILs) for a total population size of 5600. However, family 46 (PI 507681B x IA3023) was not included in our analysis because parentage inconsistency analysis using the marker data of the parents and progeny found that the cross was not as described (Song et al. 2017), leaving 39 families for this study. Moreover, the number of RILs per family was not always equal to 140 as originally intended. Number of phenotyped RILs per family ranged from 98 to 140 (Supplementary Table 1), with the total used for estimate of parameters using phenotypic data being 5174.

The relative maturity of the RILs range from late MG II to early MG IV. Due to the large family size, the RILs from each family were divided into four sets of 35. Each set was randomly assigned to an incomplete block within environment that was augmented with the two parents and three additional checks, totaling 40 entries per set and a total of 160 sets (Diers et al. 2018). The two parents and three checks were added to provide estimates of block effects and adjust the genotypic values for agronomic traits. These parents and checks were common to all environments (Supplementary Fig. 1). All RILs were assessed for seed yield in at least eight different environments (site x year combination) during the 2011–2013 period and a subset of the RILs was evaluated for agronomic traits in up to 18 environments (Diers et al. 2018). Genotypes were grown in two-row plots four m length with 0.76 m between rows.

Seven agronomic and seed composition traits were measured: seed yield (kg ha^−1^ on a 130 g kg^−1^ moisture basis), plant height (centimeters), days to maturity (number of days after planting), lodging (score 1–5), seed size (mass of 100 seed in grams); oil and protein content (% on a 130 g kg^−1^ moisture basis). The genotypic and phenotypic data as well as descriptive information of the parents are publicly available at SoyBase (https://www.soybase.org/SoyNAM). A repository containing all the scripts and documentation to reproduce the analysis is available at: https://github.com/cleitonwartha/CrossPred.

### Genotypic data

Parents and RILs were genotyped using single nucleotide polymorphism (SNP) markers with the SoyNAM6K BeadChip (Song et al. 2017). 4312 out of 5303 markers included on the SoyNAM6K BeadChip were defined by the SoyNAM project group as “quality assured” based on proportion of missing loci and correct segregation (Song et al. 2017). Marker data were obtained from the R package SoyNAM (Xavier et al. 2022). Additional information about the genetic characterization of the SoyNAM and details on how the SoyNAM6K BeadChip was designed were provided in Song et al. (2017). A small number of RILs per family did not have genotype data made available. The number of genotyped RILs per family is presented in Supplementary Table 1.

### Analysis of phenotypic data

Best linear unbiased estimates (BLUEs) of genotype effects were obtained using the following linear mixed model on the raw phenotypic data collected as described above:

1 $${y}_{{ijk}}={{\mu }}+{g}_{i}+{t}_{j}+{c}_{k}+{\varepsilon }_{{ijk}}$$

where *y* is the measured phenotypic value for a given trait, *µ* is the intercept, ${g}_{i}$ is the fixed effect of the *i*th genotype, ${t}_{j}$ is the fixed effect of the *j*th environment, ${c}_{k}$ is the fixed effect of the covariate of the family parents and check varieties in each set, and ${\varepsilon }_{{ijk}}$ is the associated residual error which was assumed to be normally and independently distributed. These BLUEs were then used as the vector of response variable in the genomic prediction (GP) models subsequently described to avoid “double-shrinkage” of values (Piepho et al. 2012).

### Prediction of genetic parameters describing value of crosses

A leave-one-family-out cross-validation scheme was implemented to form training and validation sets to assess predictive ability. Briefly, the genetic parameters (mean, genetic variance, superior progeny mean, and genetic correlation) of family *i* were predicted using all remaining individuals as the training set, except the individuals belonging to family *i*. This procedure was repeated until predictions were obtained for all families. The genotype BLUEs returned from model (1) and associated genotypic data were used as the training set to estimate marker effects using the ridge-regression best linear unbiased prediction (RR-BLUP) model:

2 $${\mathbf{y}}={\mathbf{1}}\mu +{\mathbf{Zu}}+{\boldsymbol{\varepsilon }}$$

where **y** is the vector of genotype BLUEs, µ is the overall mean, **Z** is an incidence matrix of marker genotypes coded as −1, 0, or 1 (i.e., homozygous for the reference allele, heterozygous, homozygous for the alternate allele, respectively), **u** is a vector of random marker effects where ${\bf{u}} \sim N(0,{\bf{I}}{\sigma }_{u}^{2})$, and **ε** is vector of residuals where ${\boldsymbol{\varepsilon }} \sim N\left(0,{\bf{I}}{\sigma }_{\varepsilon }^{2}\right)$.

Predicted marker effects were then used to calculate a predicted family mean ($\hat{\mu }$), genetic variance (${\hat{\sigma }}_{g}^{2}$), superior progeny mean (${\hat{\mu }}_{{sp}}$), and genetic correlation (${\hat{r}}_{g}$) for each validation set. The equations used for this purpose were analytically derived and described by Zhong and Jannink (2007) and Neyhart et al. (2019). Briefly, the equations are based on modeling of the segregation and recombination of genome-wide markers accounting for the additive effects and recombination fraction among different loci influencing a quantitative trait. Effects of genome-wide markers in linkage disequilibrium with QTL estimated from GP models are used as surrogates of the QTL effects (Meuwissen et al. 2001). Information on the recombination fraction between the different loci was retrieved from the composite linkage map across all SoyNAM families created by Song et al. (2017). The R package PopVar (Mohammadi et al. 2015) was used to implement the equations to calculate $\hat{\mu }$, ${\hat{\sigma }}_{g}^{2}$, ${\hat{\mu }}_{{sp}}$, and ${\hat{r}}_{g}$. The analytically derived equations have been shown to generate very similar results to stochastic simulations (Neyhart et al. 2019; Schopp et al. 2017).

### Estimation of genetic parameters from validation families

Statistical analysis on raw phenotypic data was conducted to obtain empirical estimates of the mean (μ), genetic variance (${\sigma }_{G}^{2}$), superior progeny mean (${\mu }_{{SP}}$), and genetic correlation (${r}_{G}$) for each family to validate the predicted values described above. A family mean was estimated by simply calculating the mean of all BLUEs of RILs belonging to that family. The superior progeny mean was calculated as the mean of the highest 10% of RIL BLUEs for oil, protein, seed size and seed yield, while the lowest 10% of RIL BLUEs were used for days to maturity, lodging, and plant height. To estimate the genetic variance of each family, we divided the dataset by family and used the following mixed linear model:

3 $${y}_{{ijk}}={{\mu }}+{c}_{k}+{g}_{i}+{t}_{j}+{\varepsilon }_{{ijk}}$$

where ${y}_{{ijk}}$ is the phenotypic value for a given trait; µ is the family mean; ${g}_{i}$ is the random effect of the *i*th genotype where ${g}_{i}$ ~ N(0, ${\sigma }_{{\rm{G}}}^{2}$); ${t}_{j}$ is the random effect of the *j*th environment where ${t}_{j}$ ~ N(0, ${\sigma }_{{\rm{t}}}^{2}$); ${c}_{k}$ is the fixed effect of the covariate family parents and check varieties in each set; and ${\varepsilon }_{{ijk}}$ is the model residual (normally and identically distributed). The variance components were estimated via restricted maximum likelihood (REML) using the average information (AI) algorithm (Gilmour et al. 1995) implemented in ASReml-R version 4.2 (VSNI, United Kingdom)(Butler et al. 2023). Standard errors were computed from the square root of the diagonal element of the inverse of the Average Information matrix (ASReml-R Reference Manual Version 4.2, p. 112). The significance of the genotypic source of variation was tested using a likelihood ratio test (LRT) between the full model from Eq. (3) and the reduced model without the genotypic source of variation. The model described in Eq. (3) was also used to compute the phenotypic reliability (*i*^2^) on a RIL-mean basis as

$${i}^{2}=1-\frac{{V}_{{BLUP}{{\_}}{diff}}}{2{\sigma }_{G}^{2}}$$

where ${V}_{{BLUP\_diff}}$ is the mean variance of a difference between a pair of genotype best linear unbiased predictions (Cullis et al. 2006).

To estimate genetic correlations between traits, the dataset was divided by family and fit to the following bi-variate mixed linear model:

4 $${\mathbf{y}}_{\mathbf{p}}={{\mathbf{1}}{{\mu }}}_{\mathbf{p}}+{\mathbf{c}}_{\mathbf{p}}+\,{\mathbf{g}}_{\mathbf{p}}+{\mathbf{t}}_{p}+{\boldsymbol{\varepsilon }}_{p}$$

where ${{\boldsymbol{y}}}_{p}=\{{y}_{{ijkp}}\}$ is the measured phenotypic value for the *p*th trait, ${{{\mu }}}_{{\boldsymbol{p}}}$ is the family mean for trait *p*, ${{\boldsymbol{g}}}_{p}=\{{g}_{{ip}}\}$ is the random effect of the *i*th genotype, ${{\boldsymbol{t}}}_{p}=\{{t}_{{jp}}\}$ is the random effect of the *j*th environment, ${{\boldsymbol{c}}}_{p}=\{{c}_{{kp}}\}$ is the fixed effect of the covariate family parents and check varieties in each set, and ${{\boldsymbol{\varepsilon }}}_{p}=\{{\varepsilon }_{{ijkp}}\}$ is the associated residual error. The model assumes a multivariate normal distribution of random effects, with ***g*** ~ MVN(0, **I** ⊗ **G**), ***t*** ~ MVN(0, **I** ⊗ **E**), and ***ε*** ~ MVN(0, **I** ⊗ **R**), where **I** is an identity matrix and ⊗ indicates the Kronecker product between matrices. The covariance structures of the genotype, environment, genotype-environment interaction, and residuals are as follow:

$${\bf{G}}=\left[\begin{array}{cc}{\sigma }_{G(1)}^{2} & {\sigma }_{G(1,2)}\\ {\sigma }_{G(2,1)} & {\sigma }_{G(2)}^{2}\end{array}\right]$$

$${\bf{E}}=\left[\begin{array}{cc}{\sigma }_{t(1)}^{2} & {\sigma }_{t(1,2)}\\ {\sigma }_{t(2,1)} & {\sigma }_{t(2)}^{2}\end{array}\right]$$

and

$${\bf{R}}=\left[\begin{array}{cc}{\sigma }_{\varepsilon (1)}^{2} & {\sigma }_{\varepsilon (1,2)}\\ {\sigma }_{\varepsilon (2,1)} & {\sigma }_{\varepsilon (2)}^{2}\end{array}\right]$$

The genetic correlation was computed from elements in **G** using the equation ${r}_{G(\mathrm{1,2})}=\frac{{\sigma }_{G(\mathrm{1,2})}}{\sqrt{{\sigma }_{G(1)}^{2}\,{\sigma }_{G(2)}^{2}}}$. Similarly to the univariate models used to estimate the genetic variance, the bi-variate models were fitted via REML using the AI algorithm implemented in the ASReml-R version 4.2 software (VSNI, United Kingdom) (Butler et al. 2023). Fisher’s transformation was used to calculate 95% confidence intervals for the correlation coefficients (Fisher 1915).

### Predictive ability (${{\boldsymbol{r}}}_{{\boldsymbol{MP}}}$) of genetic parameters

The predictive ability (${r}_{{MP}}$) for the mean, genetic variance, superior progeny mean, and genetic correlation in each validation family was measured by the Pearson’s correlation coefficient between the genomic prediction and the phenotype-based observation of each genetic parameter. Estimates of predictive ability were based on 39 validation families evaluated for all seven traits. A bootstrapping procedure using 10,000 bootstrapped samples was used to test if the correlation coefficient estimates were significantly different than zero. A 95% confidence interval was obtained by using the 250th lowest value as the lower bound and the 9750^th^ value as the upper bound of the interval. In addition, we calculated the statistical bias of the predicted genetic variance as ${(\bar{\sigma }}_{\hat{g}}^{2}-{\bar{\sigma }}_{G}^{2})\,/{\bar{\sigma }}_{G}^{2}$, where ${\bar{\sigma }}_{\hat{g}}^{2}$ was the mean predicted genetic variance and ${\bar{\sigma }}_{G}^{2}$ was the mean estimated genetic variance across all validation families (Lian et al. 2015). Negative bias indicates that the predicted values are on a lower scale than the observed values, while positive bias denotes that the predicted values are on higher scale than the observed values.

## Results

### Observed means, genetic variances, and genetic correlations

**Fig. 1:**
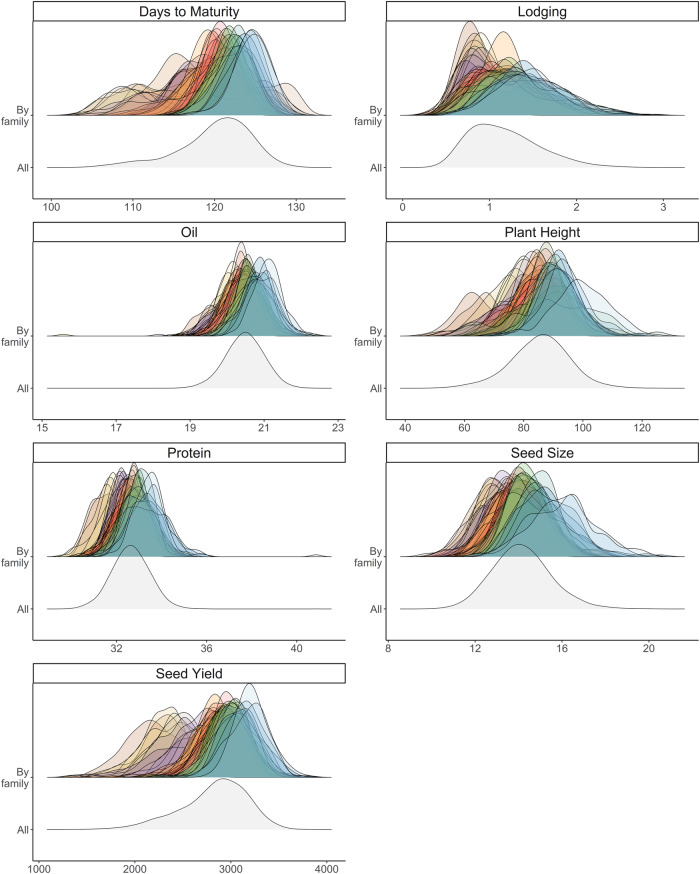
Distribution of best linear unbiased estimates of recombinant inbred line (RIL) effects for each trait. Distributions are presented for each family (top) and the overall distribution including all RILs in the SoyNAM population is presented in gray (bottom). Units for each trait are presented in Table 1.

**Fig. 2:**
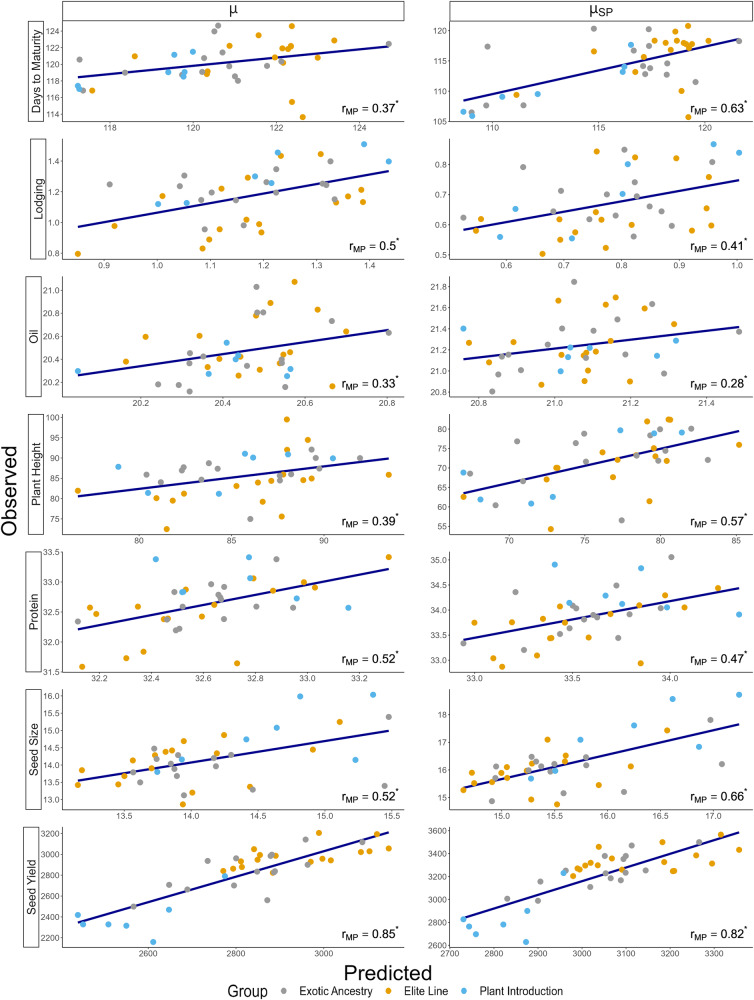
Estimates of predictive ability (*r*_*MP*_) obtained from the correlation between marker-predicted and observed values for family mean (μ) and superior progeny mean (μ_*SP*_) across 39 validation families and seven soybean traits. Each point in the scatterplot represents the value of the genetic parameter for a single validation family and was colored according to the genetic background of the family: elite (yellow), exotic ancestry (gray), and plant introduction (blue). The blue line indicates the fitted linear regression line. Asterisks after the ${r}_{{MP}}$ estimates indicate that they are significant at the 0.05 probability level (bootstrapping with 10,000 samples).

**Fig. 3:**
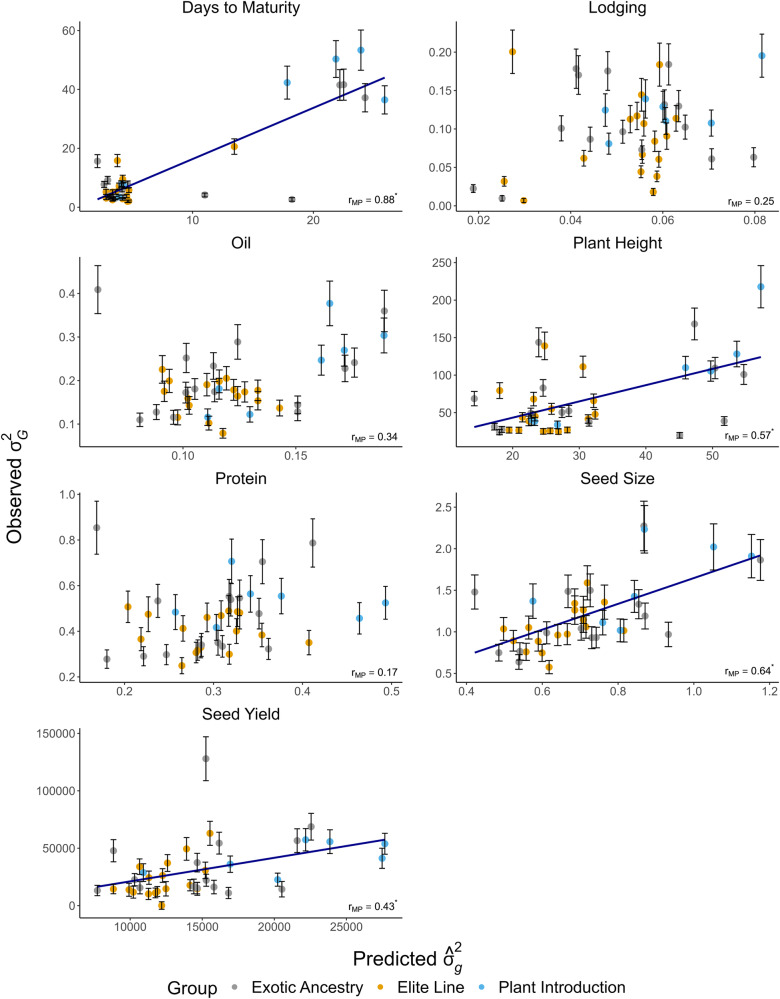
Estimates of predictive ability (r_*MP*_) obtained from the correlation between marker-predicted and observed values for genetic variance $({\sigma }_{G}^{2})$ across 39 validation families and seven soybean traits. Each point in the scatterplot represents the genetic variance value for a single validation family and was colored according to the genetic background of the family: elite (yellow), exotic ancestry (gray), and plant introduction (blue). Error bars depict the standard error for observed ${\sigma }_{G}^{2}$. The blue line indicates the fitted linear regression line. Asterisks after the ${r}_{{MP}}$ estimates indicate that they are significant at the 0.05 probability level (bootstrapping with 10,000 samples).

The 39 families comprising the SoyNAM population displayed differences in both means and variances for all traits (Fig. 1; Table 1). As expected, mean seed yield per family was uniformly highest in families with “elite” line parents bred for seed yield, lowest in families originating from crosses involving a plant introduction, and intermediate in families involving breeding lines with exotic ancestry (Fig. 2). Genetic variance was significantly different than zero for all traits in all families (*P* < 0.001). There was a large range for ${\sigma }_{G}^{2}$ among families for all traits, especially for days to maturity, plant height, and seed yield (Table 1). Despite the large family sizes (*N* = 140) and number of environments in the dataset, the standard errors and confidence intervals for ${\sigma }_{G}^{2}$ were large for some families (Fig. 3). The magnitude of the confidence intervals varied across different families assessed for different soybean traits. Estimated phenotypic reliabilities (*i*^2^) on an entry-mean basis were generally high, but varied among traits and families, with mean (and range) values varying from 0.55 (0.32–0.82) for seed yield to 0.92 (0.88–0.95) for seed size (Table 1).

**Table 1:** Descriptive analysis of the phenotypic data: mean (and range) of the observed family mean (*μ*), genetic variance (${\sigma }_{G}^{2}$), superior progeny mean (${\mu }_{{SP}}$), and phenotypic reliability (${i}^{2}$) for seven soybean traits measured on recombinant inbred lines from 39 families comprising the Soybean Nested Association Mapping Population.

Trait	*μ*	${\sigma }_{G}^{2}$	${\mu }_{{SP}}$	${i}^{2}$^b^
Days to maturity (DAP^a^)	120.1 (113.64; 124.66)	12.8 (2.0; 53.3)	114.4 (105.7; 120.8)	0.86 (0.72; 0.97)
Lodging (1–5 scale)	1.2 (0.8; 1.5)	0.1 (0.01; 0.2)	0.7 (0.5; 0.9)	0.75 (0.34; 0.87)
Oil (%)	20.5 (20.1; 21.1)	0.2 (0.08; 0.4)	21.2 (20.8; 21.8)	0.90 (0.83; 0.95)
Plant Height (cm)	85.6 (72.5; 99.5)	65.9 (19.7; 217.9)	71.6 (54.3; 82.5)	0.90 (0.82; 0.96)
Protein (%)	32.6 (31.6; 33.4)	0.5 (0.2; 0.9)	33.9 (32.9; 35.1)	0.88 (0.79; 0.95)
Seed Size [g (100 seed)^−1^]	14.2 (12.8; 16.0)	1.2 (0.6; 2.3)	16.2 (14.7; 18.7)	0.92 (0.88; 0.95)
Seed Yield (kg ha^−1^)	2823.8 (2159.4; 3205.3)	31,302.8 (77.3; 127,933.7)	3210.9 (2628.7; 3565.3)	0.55 (0.32; 0.82)

^a^*DAP* days after planting.

^b^Phenotypic reliability computed on an RIL-mean basis according to Cullis et al. (2006).

Family-wise estimates of genetic correlations varied considerably both across pairs of traits, as well as across families for any given pair of traits (Table 2). The strongest positive *r*_*G*_ estimated was 0.65 between plant height and lodging, with a range from 0.37 to 0.84 across families. The strongest negative *r*_*G*_ estimated was −0.58 between oil and protein, with a range from −0.28 to −0.76 across families. The mean *r*_*G*_ between protein and seed yield, which are well known to be negatively correlated, was −0.12. The estimates of *r*_*G*_ for protein and yield across families ranged from −0.55 to 0.28, with very few families exhibiting a positive estimate of *r*_*G*._

**Table 2:** Mean and range (in parentheses) of observed genetic correlations (${r}_{G}$).

Trait 1	Trait 2	${r}_{G}$	${r}_{{MP}}$(${\hat{r}}_{g},\,{r}_{G}$)
Days to Maturity	Lodging	0.36 (−0.14; 0.78)	0.53* (0.27; 0.73)
Days to Maturity	Oil	−0.32 (−0.72; 0.13)	0.59* (0.34; 0.76)
Days to Maturity	Plant Height	0.55 (0.26; 0.88)	0.47* (0.15; 0.70)
Days to Maturity	Protein	0.08 (−0.33; 0.37)	0.18 (−0.11; 0.46)
Days to Maturity	Seed Size	0.00 (−0.54; 0.36)	0.68* (0.47; 0.83)
Days to Maturity	Seed Yield	0.31 (−0.21; 0.76)	0.46* (0.15; 0.71)
Lodging	Oil	−0.18 (−0.62; 0.23)	0.52* (0.17; 0.77)
Lodging	Protein	0.02 (−0.22; 0.28)	0.27 (−0.05; 0.54)
Lodging	Seed Size	−0.14 (−0.45; 0.21)	0.57* (0.35; 0.74)
Lodging	Seed Yield	−0.22 (−0.75; 0.36)	0.54* (0.34; 0.71)
Oil	Protein	−0.58 (−0.76; −0.28)	0.27* (0.06; 0.52)
Oil	Seed Size	0.17 (−0.11; 0.37)	0.32* (0.06; 0.54)
Oil	Seed Yield	0.02 (−0.43; 0.50)	0.55* (0.34; 0.73)
Plant Height	Lodging	0.65 (0.37; 0.84)	0.52* (0.20; 0.72)
Plant Height	Oil	−0.23 (−0.64; 0.2)	0.68* (0.41; 0.86)
Plant Height	Protein	0.07 (−0.21; 0.36)	0.30* (0.02; 0.54)
Plant Height	Seed Size	−0.06 (−0.46; 0.35)	0.59* (0.32; 0.78)
Plant Height	Seed Yield	−0.03 (−0.52; 0.64)	0.56* (0.32; 0.76)
Protein	Seed Size	0.09 (−0.26; 0.46)	0.61* (0.46; 0.73)
Protein	Seed Yield	−0.12 (−0.55; 0.28)	0.26 (−0.05; 0.56)
Seed Size	Seed Yield	0.13 (−0.32; 0.63)	0.56* (0.36; 0.73)

Estimates of predictive ability (${{\boldsymbol{r}}}_{{\boldsymbol{MP}}}$; 95% confidence interval in parentheses) measured as the correlation between the marker-predicted and observed genetic correlation (${{\boldsymbol{r}}}_{{\boldsymbol{G}}}$) for each of the 21 soybean trait combinations. The 95% confidence interval of the predictive ability was estimated from 10,000 bootstrapping samples.

^*^Significant at *P* < 0.05, bootstrapping.

### Predictive ability of family means, genetic variances, and genetic correlations

We evaluated the predictive ability of genomic prediction models for family mean, genetic variance, and trait correlations using a leave-one-family-out cross validation scheme (Table 3). The predictive ability for family mean (*µ*) was significantly greater than zero (*P* < 0.05) for all traits. According to general guidelines for the strength of a correlation (Cohen 1988), the ${r}_{{MP}}$ for *µ* was high (0.50 ≤ ${r}_{{MP}}$ < 1.0) for most traits, while it was moderate (0.30 ≤ ${r}_{{MP}}$ < 0.50) for oil, days to maturity, and plant height. Meanwhile, the predictive ability for the genetic variance (${\sigma }_{G}^{2}$) was significant (*P* < 0.05) for four of the seven traits studied (Table 3; Fig. 3). The correlation between observed ${\sigma }_{G}^{2}$ and predicted genetic variance was not significant for protein, lodging, and oil. However, the protein and oil distributions of BLUEs for family 25 exhibited high degrees of skewness and kurtosis (Supplementary File 1). This family also showed a large deviation between the predicted and observed genetic variance for oil and protein, with the point representing this family being in the upper left-hand corner of the scatter plot (oil and protein panels in Fig. 3). If this family is removed from the analysis, then the predictive ability for the genetic variance goes from being non-significant (0.34 and 0.17 for oil and protein, respectively) to significantly different than zero (0.59 and 0.39 for oil and protein, respectively).

**Table 3:** Estimates of predictive ability (${r}_{{MP}}$) measured as the correlation between the marker-predicted and observed values for family mean (*μ*), genetic variance (${{\boldsymbol{\sigma }}}_{{\boldsymbol{G}}}^{{\boldsymbol{2}}}$), and superior progeny mean (${{\boldsymbol{\mu }}}_{{\boldsymbol{SP}}}$) for seven soybean traits.

Trait	${r}_{{MP}}$($\hat{\mu },\,$^*µ*^)	${r}_{{MP}}$(${\hat{\sigma }}_{g}^{2},\,{\sigma }_{G}^{2}$)	${r}_{{MP}}$(${\hat{\mu }}_{{sp}},\,{\mu }_{{SP}}$)	${\hat{\sigma }}_{g}^{2}$ ^Bias (%)^
Days to Maturity	0.37* (0.04; 0.70)	0.88* (0.71; 0.96)	0.63* (0.31; 0.85)	−37.9
Lodging	0.50* (0.25; 0.69)	0.25 (−0.21; 0.61)	0.41* (0.14; 0.64)	−47.9
Oil	0.33* (0.08; 0.54)	0.34 (−0.14; 0.73)	0.28* (0.02; 0.52)	−36.4
Plant Height	0.39* (0.19; 0.58)	0.57* (0.24; 0.79)	0.57* (0.37; 0.75)	−53.8
Protein	0.52* (0.24; 0.73)	0.17 (−0.23; 0.56)	0.47* (0.2; 0.70)	−32.4
Seed Size	0.52* (0.18; 0.77)	0.64* (0.37; 0.80)	0.66* (0.41; 0.83)	−40.6
Seed Yield	0.85* (0.75; 0.91)	0.43* (0.22; 0.71)	0.82* (0.71; 0.89)	−52.2

A 95% confidence interval (in parentheses) for the predictive ability was estimated from 10,000 bootstrapping samples.

^*^Significant at *P* < 0.05, bootstrapping.

**Fig. 4:**
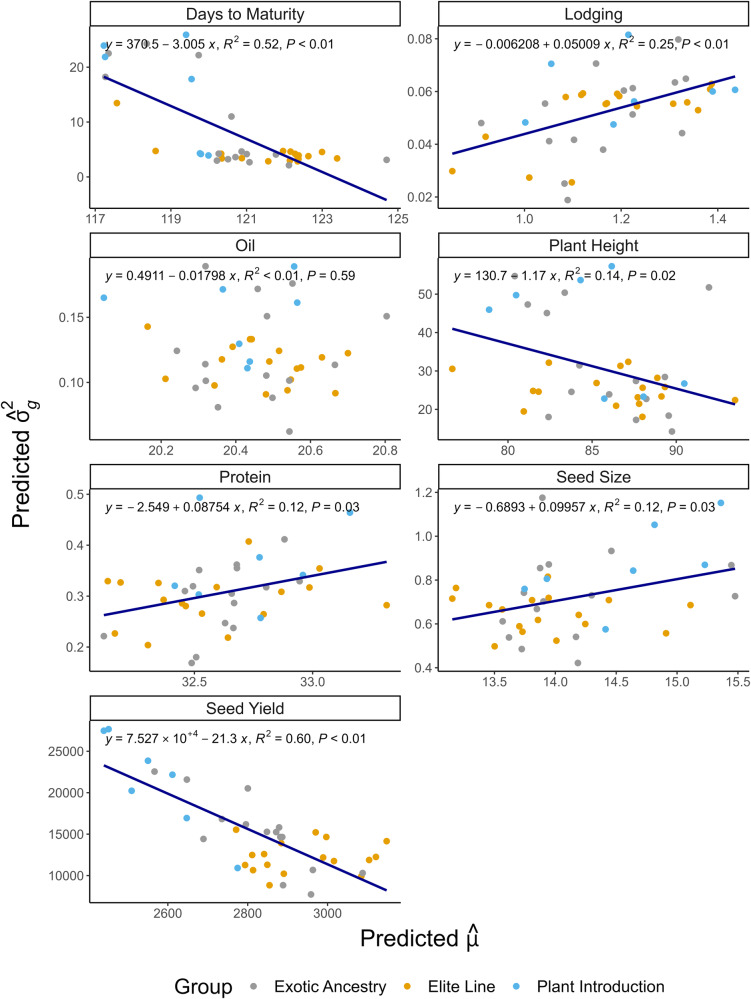
Scatterplot of predicted genetic variance $({\widehat{\sigma }}_{g}^{2})$ plotted against the predicted family means $(\widehat{\mu })$ of 39 validation families for seven soybean traits. Each point in the scatterplot represents the value of the genetic parameter for a single validation family and was colored according to the genetic background of the family: elite (yellow), exotic ancestry (gray), and plant introduction (blue). The blue line indicates the fitted linear regression line.

When compared to the mean observed ${\bar{\sigma }}_{G}^{2}$ across all validation families, the mean predicted ${\bar{\sigma }}_{\hat{g}}^{2}$ displayed a downward bias ranging from −32% for protein to −54% for plant height (Table 3). This moderate negative bias observed on genetic variance predictions was consistent across validation families and may have little impact on choice of breeding crosses. We also observed a negative covariance between predicted $\hat{\mu }$ and ${\hat{\sigma }}_{g}^{2}$ for days to maturity, plant height, and seed yield (Fig. 4). A negative covariance between observed family *µ* and ${\sigma }_{G}^{2}$ was also found for days to maturity, oil, and seed yield (Supplementary Fig. 2).

Predictive ability for the family superior progeny mean (${\mu }_{{SP}}$) was significantly different from zero (*P* < 0.05, bootstrapping) for all traits. Given the relative contribution of both the *µ* and ${\sigma }_{G}^{2}$ to the ${\mu }_{{SP}}$, the predicted ability for ${\mu }_{{SP}}$ was expected to have an intermediate value between *µ* and ${\sigma }_{G}^{2}$. However, higher predictive abilities for ${\mu }_{{SP}}$ as compared to *µ* were observed for seed size, days to maturity, and plant height due to the influence of the larger predictive abilities observed for ${\sigma }_{G}^{2}$ than for *µ* for these same traits.

**Fig. 5:**
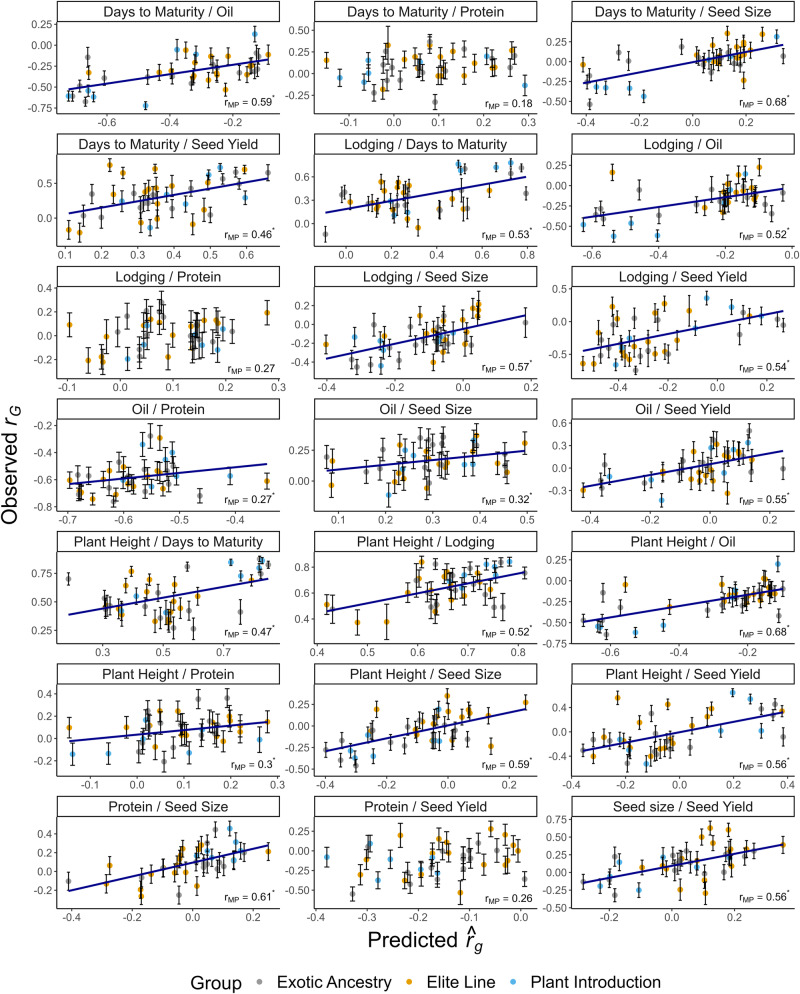
Estimates of predictive ability (*r*_*MP*_) obtained from the correlation between marker-predicted and observed values for genetic correlation (*r*_*G*_) across 39 validation families and 21 soybean trait combinations. Each point in the scatterplot represents the genetic correlation value for a single validation family and was colored according to the genetic background of the family: elite (yellow), exotic ancestry (gray), and plant introduction (blue). Error bars depict the 95% confidence interval for observed ${r}_{G}$. The blue line indicates the fitted linear regression line. Asterisks after the ${r}_{{MP}}$ estimates indicate that they are significant at the 0.05 probability level (bootstrapping with 10,000 samples).

The predictive ability of ${r}_{G}$ was significant for 18 (86%) out of the 21 pairwise trait combinations (*P* < 0.05) (Table 2; Fig. 5). Among those that were found to be significantly different than zero, predictive ability was low (${r}_{{MP}}$ < 0.30) for one trait combination, moderate (0.30 ≤ ${r}_{{MP}}$ < 0.50) for four trait combinations, and high (0.50 ≤ ${r}_{{MP}}$ < 1.0) for 13 trait combinations (Table 2). The nonsignificant predictive abilities in this study were observed for the trait pairs protein and days to maturity, protein and seed yield, and protein and lodging.

## Discussion

The aim of this study was to test genomic prediction of family means, genetic variances, superior progeny means, and genetic correlations for soybean traits. To accomplish this we used existing data from the SoyNAM population (Diers et al. 2018) which consists of a large number of families (39) and many progenies per family ( ~ 140). Osthushenrich et al. (2018) discussed the limitations in performing a pure validation study and the limited transfer of these results to practical breeding programs because of differences in the experimental setup. Limitations of the SoyNAM for this empirical validation are the close relatedness of the training population (TP) to the validation families (Habier et al. 2007; Riedelsheimer et al. 2013), since the validation families shared the common parent IA3023 with the families used in the TP, and the diverse background of founders (elite, breeding lines with exotic ancestry, and plant introductions). Although SoyNAM was not specifically designed for a rigorous validation of cross selection through genomic prediction, the amount and quality of the data from this publicly available resource makes it worthy for an initial validation experiment. The vast majority of studies on the prediction of genetic variance have used simulations, and we are not aware of any studies using real data to predict genetic correlations between traits in soybean. Our results, although possibly overly optimistic, warrant the design of further validation experiments that better resemble real-life breeding scenarios.

Validating the predictions of ratios of second-order statistics such as genetic correlations requires large family sizes because estimates are subject to large sampling variances (Robertson 1959). As noted in the *Results* section, the standard errors and confidence intervals for ${\sigma }_{G}^{2}$ were large for some families (Fig. 3). In addition, genetic variance estimates for oil and protein content were small in comparison to previously reported values in the literature (Mourtzinis et al. 2017; Assefa et al. 2019). The SoyNAM founder lines were not selected on the basis of variation for seed composition, but rather for seed yield, diverse ancestry, or drought tolerance (Song et al. 2017).

The previously reported negative protein-seed yield and protein-oil correlations (Hartwig and Hinson 1972; Wehrmann et al. 1987; Wilcox and Shibles 2001) were also found in this study although lower in magnitude (Table 2). The unfavorable ${r}_{G}$ between protein and seed yield imposes a challenge to breeders and has limited progress in the development of soybean cultivars with both high yield and protein (Rincker et al. 2014; Wilson 2004). Days to maturity was positively correlated with seed yield and plant height, being consistent with results from previous studies (Cicek et al. 2006). Oil and seed yield are often positively correlated (Hartwig and Hinson 1972; Wilcox and Shibles 2001), but the mean correlation estimated among SoyNAM families was nearly zero (${r}_{G}$ = 0.02). The strength and direction of mean correlations in the SoyNAM may have been affected by several factors, including the diverse genetic background of the founder lines contributing to the wide range in ${r}_{G}$ across the families (Table 2).

Our results demonstrated that the family mean for different soybean traits can be predicted for biparental families using genome-wide markers, with ${r}_{{MP}}$ estimates ranging from 0.33 to 0.85 across traits (Table 3). Moderate to high predictive ability estimates of genetic variance were observed for days to maturity, seed size, plant height, and seed yield. Moreover, the predictive abilities of protein and oil would also have been significant if one family (family 25) exhibiting skewness and kurtosis was removed from the analysis. The high predictive ability for days to maturity (${r}_{{MP}}$ = 0.88), plant height (${r}_{{MP}}$ = 0.57), and seed yield (${r}_{{MP}}$ = 0.43) are surprisingly large when compared to published empirical validation studies for analogous traits in other crops such as barley (Neyhart and Smith 2019) and maize (Adeyemo and Bernardo 2019; Lian et al. 2015).

We speculate that the larger ${r}_{{MP}}$ found for these traits in our study can be partially explained by many families with large population sizes required to handle the inherent sampling variance of these parameters. Additionally, the TP and validation families were assessed in the same set of locations and years and, therefore, any marker-by-environment interaction effects were common to the TP and VP. Moreover, the high correlations were driven by larger ${\sigma }_{G}^{2}$ values observed in families created using a plant introduction as a parent clearly exemplified by two clusters of points in the cases of days to maturity and plant height (Fig. 3). Correlation coefficients are known to be influenced by the data range (Janse et al. 2021), and the wider range of data points from families with plant introductions as parents likely inflated the ${r}_{{MP}}$ for some traits. On the other hand, we were unable to predict ${\sigma }_{G}^{2}$ for protein, lodging, and oil. The second-moment of a distribution, such as a variance, is inherently difficult to accurately predict since it can be more impacted by error in the estimates of marker effects (Zhong and Jannink 2007). Moreover, even though significant ${\sigma }_{G}^{2}$ was observed for all families for protein, oil, and lodging, the range of ${\sigma }_{G}^{2}$ variation was surprisingly small (e.g., total range of 0.5%^2^ for protein). A likely reason is that founder parents were selected on the basis of adequate plant standability, and variation in seed composition traits was not considered in the choice of the SoyNAM founders. Hence, we suspect that the pitfall described above on the upward influence of the data range on ${r}_{{MP}}$ might have impacted ${r}_{{MP}}$ for protein, oil, and lodging in the just the opposite way. Furthermore, we observed that the predictive ability of the family *µ* and ${\sigma }_{G}^{2}$ for seven soybean traits did not correlate well (*r* = −0.20). This result, also reported by Wolfe et al. (2021), suggests that a breeder cannot directly rely on estimates of ${r}_{{MP}}$ of the family *µ* as a proxy for the suitability of predicting ${\sigma }_{G}^{2}$.

Negative biases, ranging from −32 to −54%, for predicted ${\hat{\sigma }}_{g}^{2}$’s were observed for all seven soybean traits. This is consistent with previously published results (Lian et al. (2015); Adeyemo and Bernardo (2019); Neyhart and Smith 2019), and expected due to the shrinkage of marker effects proportionally to the error/signal ratio as a parameterization applied by GP models such as RR-BLUP (Lian et al. 2015). Given the relative consistency observed in bias across validation families observed in our study (Fig. 3) along with sufficient predictive ability, the observed bias and underestimation of the true ${\sigma }_{G}^{2}$ may not impact the relative ranking of crosses defined on the basis of ${\hat{\sigma }}_{g}^{2}$ or ${\hat{\mu }}_{{sp}}$. We demonstrated that the ${\mu }_{{SP}}$ can be accurately predicted from genome-wide marker effects with moderate to high predictive abilities for most traits (Table 3). In addition, we found that the predictive abilities of family *µ* and ${\mu }_{{SP}}$ for seven soybean traits were well correlated (*r* = 0.70).

A negative covariance between predicted $\hat{\mu }$ and ${\hat{\sigma }}_{g}^{2}$ was observed in the current study for days to maturity, plant height, and seed yield (Fig. 4). Zhong and Jannink (2007) described this relationship as occurring in crosses attempted between high-phenotype value RILs that are fixed at many loci for favorable alleles, yielding populations with both high $\hat{\mu }$ and low ${\hat{\sigma }}_{g}^{2}$. The grouping of families made from founder lines derived from different levels of breeding (elite, exotic ancestry, and plant introduction) allows us to evaluate this hypothesis. For seed yield, we observed that elite families exhibited a high $\hat{\mu }$ and low ${\hat{\sigma }}_{g}^{2}$, while families from a founder with exotic ancestry resulted in intermediate $\hat{\mu }$ and ${\hat{\sigma }}_{g}^{2}$ values, and families with a plant introduction as a founder had the largest ${\hat{\sigma }}_{g}^{2}$ values at the expense of low $\hat{\mu }$ (Fig. 4). The same trend was observed for days to maturity and plant height but with less defined separation of the family groups. Disregarding the differences in breeding intensity and selection that the founder lines had undergone, and considering only the 17 families derived from elite founder lines, a covariance between predicted $\hat{\mu }$ and ${\hat{\sigma }}_{g}^{2}$ was not found (data not shown).

Predictive abilities for ${r}_{G}$ were high for 13 trait combinations, and significantly greater than zero for all trait combinations except three, which all happened to include protein (Table 2). We suspect that the small amount of variation in ${\sigma }_{G}^{2}$ (e.g., total range of 0.5%^2^ for protein) likely lowered ${r}_{{MP}}$, since the range of the correlation is known to influence the correlation coefficient (Janse et al. 2021). The moderate to high ${r}_{{MP}}$ observed for the majority of trait combinations, however, demonstrates that predictions of ${r}_{G}$ are possible with reliable training data. The ${r}_{{MP}}$ for individual traits is expected to increase as the product of the TP size and the trait heritability (Nh^2^) (Daetwyler et al. 2008). In this study, the phenotypic reliability (*i*^2^) of individual traits was high, ranging from 0.55 to 0.92 (Table 1), and TP sizes were nearly 5,000 individuals in the leave-one-family-out CV scheme. Breeders desiring to implement such approaches should investigate the effect of TP size and *i*^2^ in their own datasets before application. Finally, it should be noted that genotype-by-environment interactions were not modeled in this study, and we assumed all environments form a single target population of environments (TPEs). Genotype-by-environment (GxE) interactions would influence genetic variances and correlations, and thus any training data used to predict such parameters should be collected from the environments representing the TPE, or appropriate GxE genomic prediction models should be used.

## Conclusion

A leave-one-family-out cross validation analysis on the SoyNAM suggested that genomic prediction can help breeders select parental combinations on the basis of resulting population mean, genetic variance, superior progeny mean, and genetic correlations among traits. This methodology is not very computationally intensive and may utilize marker and phenotypic data already available in programs routinely deploying genomic selection. There are several caveats to the conclusions associated with our use of the SoyNAM population structure, and thus we advocate for testing of these methods within specific breeding programs. If further testing also indicates usefulness of this methodology, it should be implemented as another tool for the breeder to enhance opportunities to find those breeding populations more likely to yield superior transgressive segregants for multiple traits.

### Supplementary information

Supplemental Figure 1

Supplemental Figure 2

Supplementary File 1

Supplementary table 1

## Data Availability

All data used in this study can be found at https://soybase.org/SoyNAM/index.php.
