# Supplementary material for: Genomic predictions of genetic variances and correlations among traits for breeding crosses in soybean
Source: Heredity (Edinb). 2024 Jul 12;133(3):173–85. doi: 10.1038/s41437-024-00703-3 (PMC11350137; doi:10.1038/s41437-024-00703-3)
Supplement: Supplementary file 1 — Supplemental Figure 1 [file 41437_2024_703_MOESM1_ESM.pdf]

Supplementary Figure 1. Heatmap displaying number of times each check, hub parent, and founder line were evaluated in each environment.

Environment

Check	2011_IL	2011_NE	2012_IA	2012_IL	2012_IN	2012_KS	2012_MI	2012_MO	2012_NE	2012_OHmc	2012_OHmian	2013_IA	2013_IL	2013_IN	2013_KS	2013_MO	2013_NE	2013_OHmc	2013_OHmian
IA3023	130	130	200	200	200	155	10	39	200	38	45	200	200	200	127	26	200	56	66
U06-100052	78	78	156	156	156	97	24	25	156	49	49	156	156	156	98	24	156	49	48
LD04-13265	78	78	120	120	120	93	10	25	120	25	22	120	120	120	78	23	120	35	32
LD04-11056	78	78	120	120	120	93	10	25	120	25	22	120	120	120	78	23	120	35	32
U09-215057			36	36	36	4	14		36	24	27	36	36	36	20	1	36	14	16
IA2094			36	36	36	4	14		36	24	27	36	36	36	20	1	36	14	16
TN05-3027	24	24	38	38	38	29		9	38	12	15	38	38	38	21	12	38	15	9
LD02-4485	2	2	40	40	40	6	15	1	40	25	29	40	40	40	23	2	40	15	17
S06-13640			4	4	4	3		1	4	1	1	4	4	4	3	1	4	1	1
PI574486	2	2	4	4	4	3		1	4	1	1	4	4	4	3		4	1	1
PI561370	2	2	4	4	4	3		1	4	1	1	4	4	4	3		4	1	1
PI427136	2	2	4	4	4	3		1	4	1	1	4	4	4	3		4	2	2
NE3001	2	2	4	4	4	3	1		4	1	1	4	4	4	3	1	4	1	1
Maverick	2	2	4	4	4	3		1	4	1	1	4	4	4	3		4	2	1
LG90-2550	2	2	4	4	4	2	1		4	1	2	4	4	4	3		4	1	1
LG05-4832	2	2	4	4	4	3		1	4	1	1	4	4	4	3		4	1	1
LG05-4317	2	2	4	4	4	3		1	4	1	1	4	4	4	3		4	2	1
LG04-4717	2	2	4	4	4	3	1	1	4	1	1	4	4	4	3		4	1	1
LG00-3372	2	2	4	4	4	3		1	4	1	1	4	4	4	2		4	1	2
LD01-5907	2	2	4	4	4	3		1	4	1	1	4	4	4	3	1	4	1	2
LD00-3309	2	2	4	4	4	3	1	1	4	1	1	4	4	4	3		4	2	1
5M20-2-5-2	2	2	4	4	4	3	1		4	1	1	4	4	4	2		4	2	1
Prohio	2	2	4	4	4	3	1		4	1	1	4	4	4	2	1	4	2	1
PI518751	2	2	4	4	4	2		1	4	1	2	4	4	4	3	1	4	1	1
PI404188A	2	2	4	4	4	2		1	4	1	2	4	4	4	3	1	4	1	1
Magellan	2	2	4	4	4	3		1	4	1	1	4	4	4	2	1	4	1	1
LG98-1605	2	2	4	4	4	2	1	1	4	1	2	4	4	4	3		4	1	2
LG05-4292	2	2	4	4	4	3	1		4	1	1	4	4	4	2	1	4	1	1
LG04-6000	2	2	4	4	4	3		1	4	1	1	4	4	4	2	1	4	1	1
LD02-9050	2	2	4	4	4	3		1	4	1	1	4	4	4	2	1	4	1	1
HS6-3976	2	2	4	4	4	2	1		4	2	1	4	4	4	3	1	4	1	1
CL0J095-4-6	2	2	4	4	4	2	1		4	1	2	4	4	4	3	1	4	1	1
4J105-3-4	2	2	4	4	4	2	1		4	2	1	4	4	4	3	1	4	1	1
U03-100612	2	2	4	4	4	2	1	1	4	1	2	4	4	4	2	1	4	2	1
Skylla	2	2	4	4	4	2	1		4	2	1	4	4	4	2		4	1	2
PI437169B	2	2	4	4	4	2	1	1	4	1	2	4	4	4	2	1	4	2	1
PI398881	2	2	4	4	4	2	1		4	2	1	4	4	4	2	1	4	1	2
LG97-7012	2	2	4	4	4	2	1	1	4	1	2	4	4	4	2	1	4	2	1
LG94-1906	2	2	4	4	4	2	1		4	2	1	4	4	4	2	1	4	1	1
LG94-1128	2	2	4	4	4	2	1		4	1	2	4	4	4	2	1	4	2	2
LG92-1255	2	2	4	4	4	2	1		4	2	1	4	4	4	2		4	1	2
LG05-4464	2	2	4	4	4	2	1		4	2	1	4	4	4	2	1	4	1	1
LG03-3191	2	2	4	4	4	2	1	1	4	2	1	4	4	4	3	1	4	1	1
LG03-2979	2	2	4	4	4	2	1	1	4	2	1	4	4	4	2	1	4	1	1
CL0J173-6-8	2	2	4	4	4	2	1	1	4	2	1	4	4	4	2		4	1	2

N Observations: 50 100 150 200
