# Supplementary material for: Genomic predictions of genetic variances and correlations among traits for breeding crosses in soybean
Source: Heredity (Edinb). 2024 Jul 12;133(3):173–85. doi: 10.1038/s41437-024-00703-3 (PMC11350137; doi:10.1038/s41437-024-00703-3)
Supplement: Supplementary file 2 — Supplemental Figure 2 [file 41437_2024_703_MOESM2_ESM.pdf]

Supplementary Figure 2. Scatterplot of observed genetic variance (σ_g^2) plotted against the observed family means (μ) of 39 validation families for seven soybean traits. Each point in the scatterplot represents the value of the genetic parameter for a single validation family and was colored according to the genetic background of the family: elite (yellow), exotic ancestry (gray), and plant introduction (blue). The blue line indicates the fitted linear regression line.

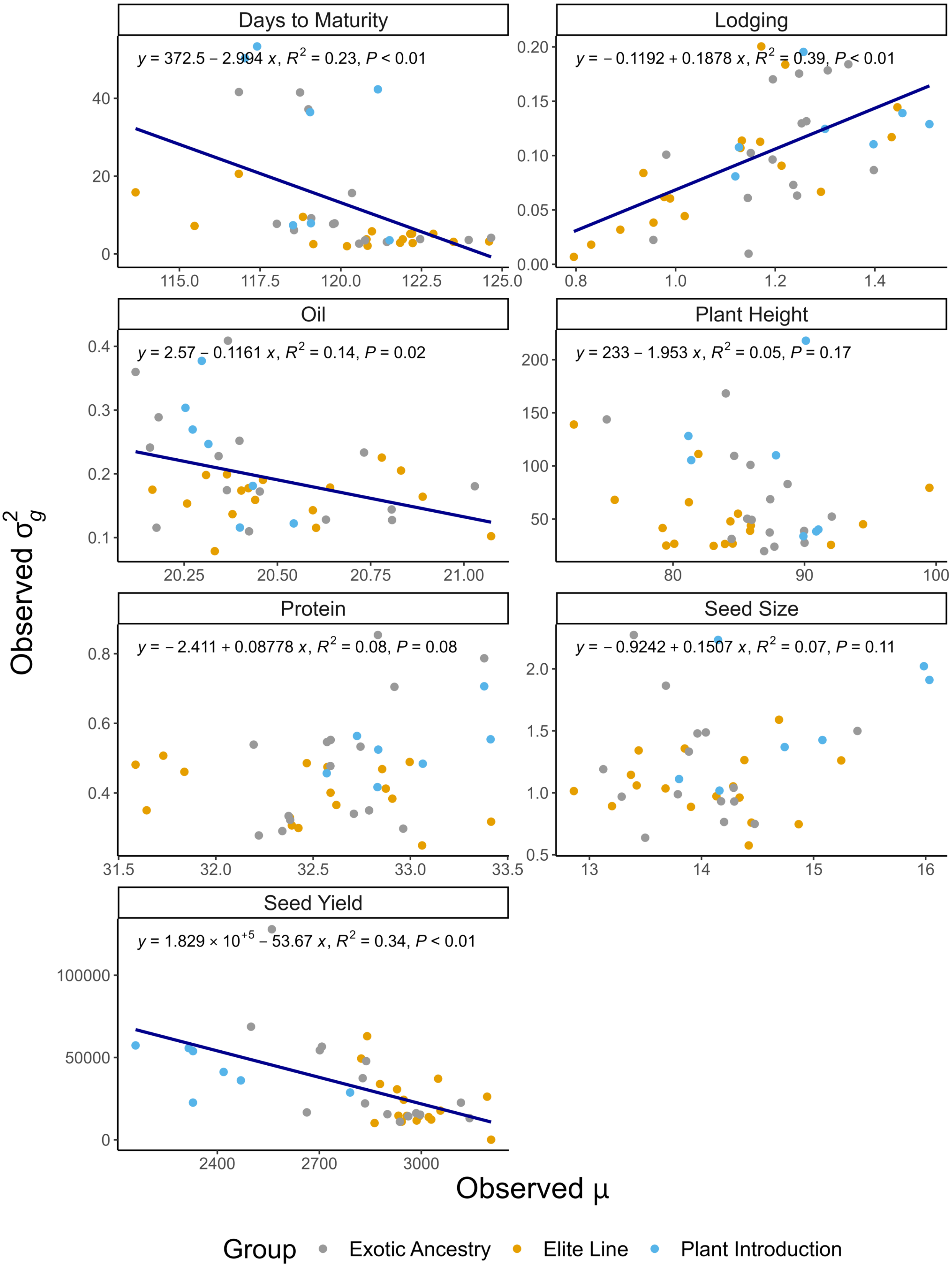

Supplementary File 1. Distribution of recombinant inbred line BLUEs for each trait within each individual family. Units for each trait are listed in Table 1. Additional metrics for normality testing skewness and kurtosis are reported.

See separate file
