# Supplementary figures and images for: Genomic predictions of genetic variances and correlations among traits for breeding crosses in soybean
Source: Heredity (Edinb). 2024 Jul 12;133(3):173–85. doi: 10.1038/s41437-024-00703-3 (PMC11350137; doi:10.1038/s41437-024-00703-3)

# Seed Yield

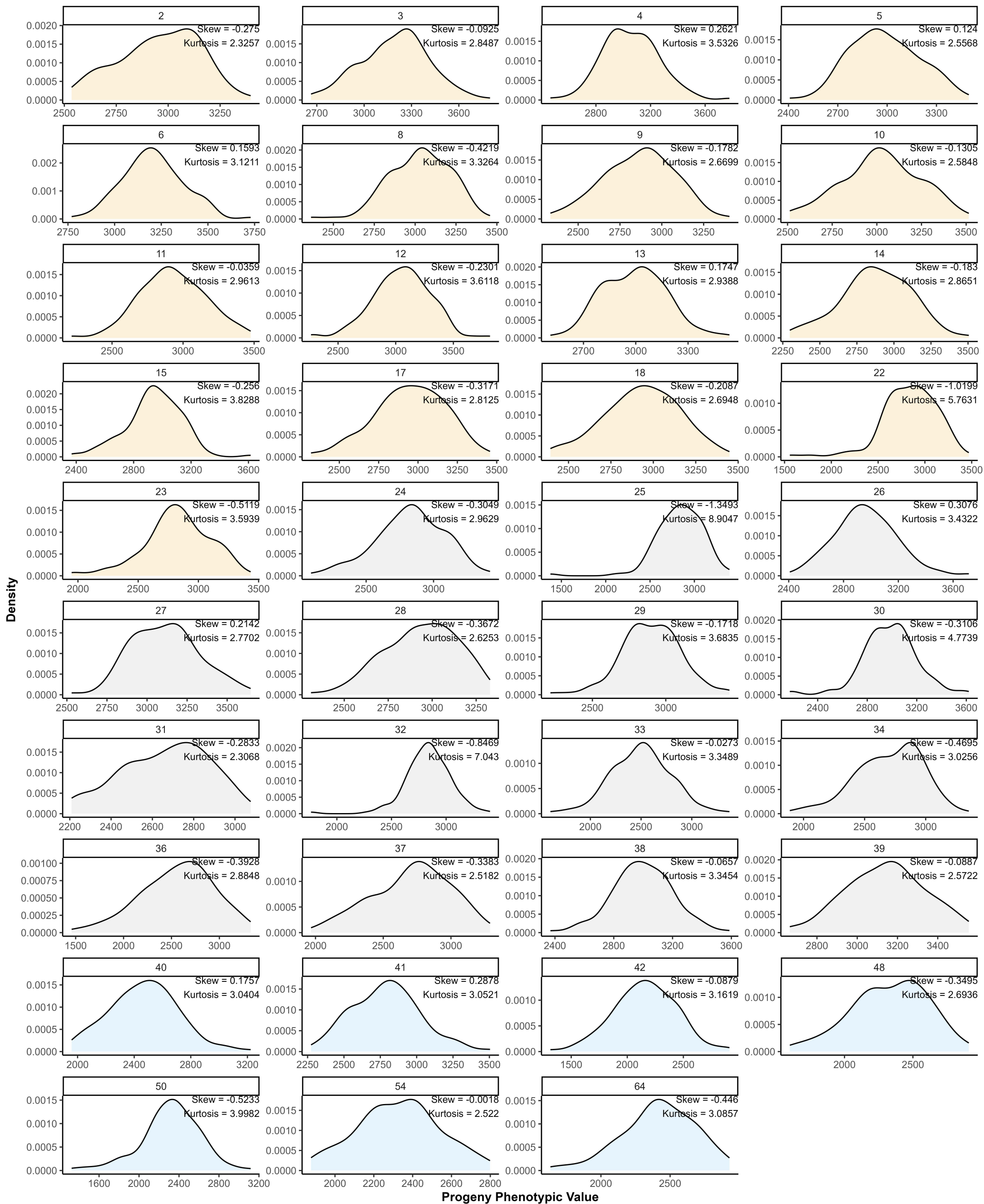

# Days to Maturity

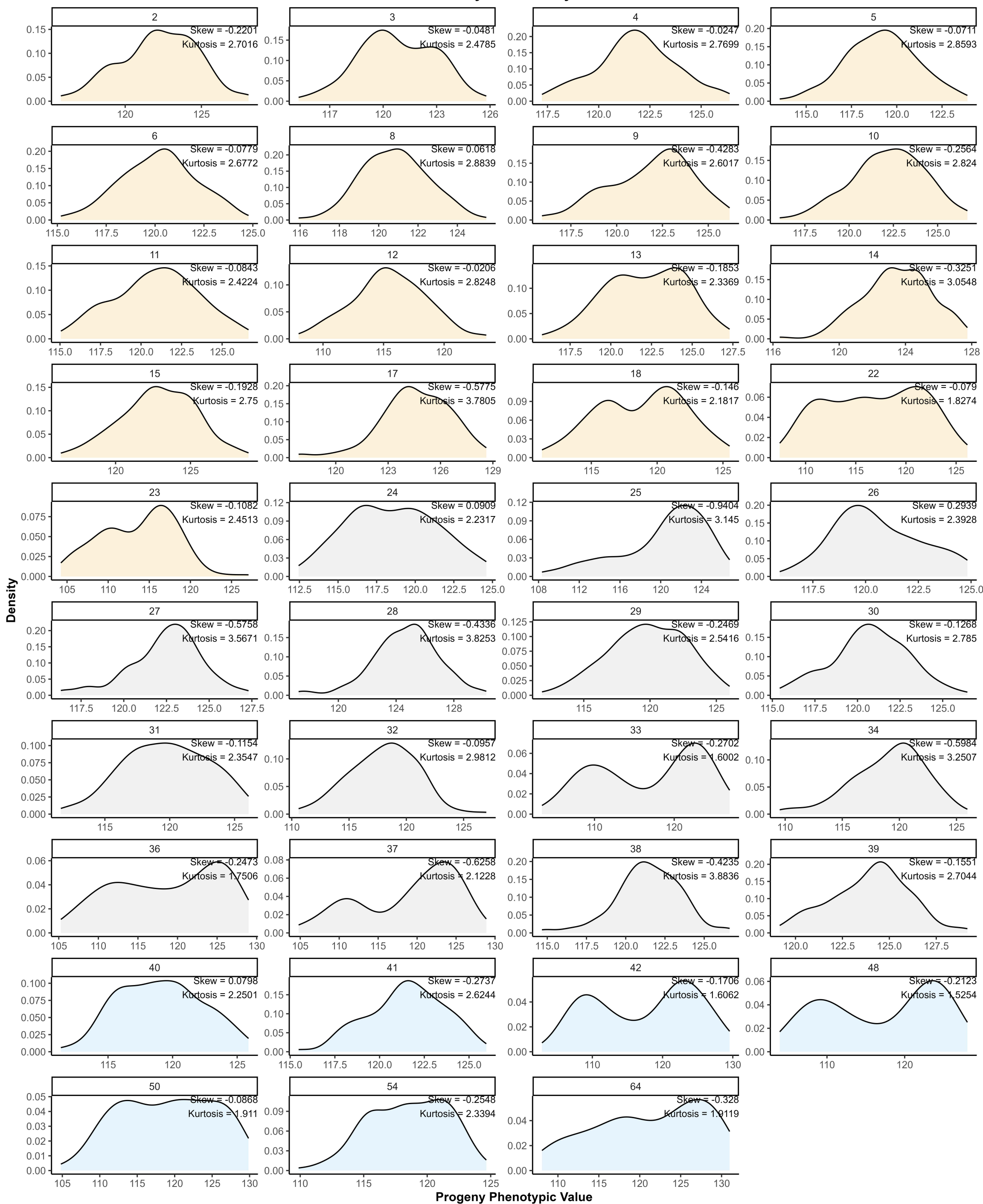

# Lodging

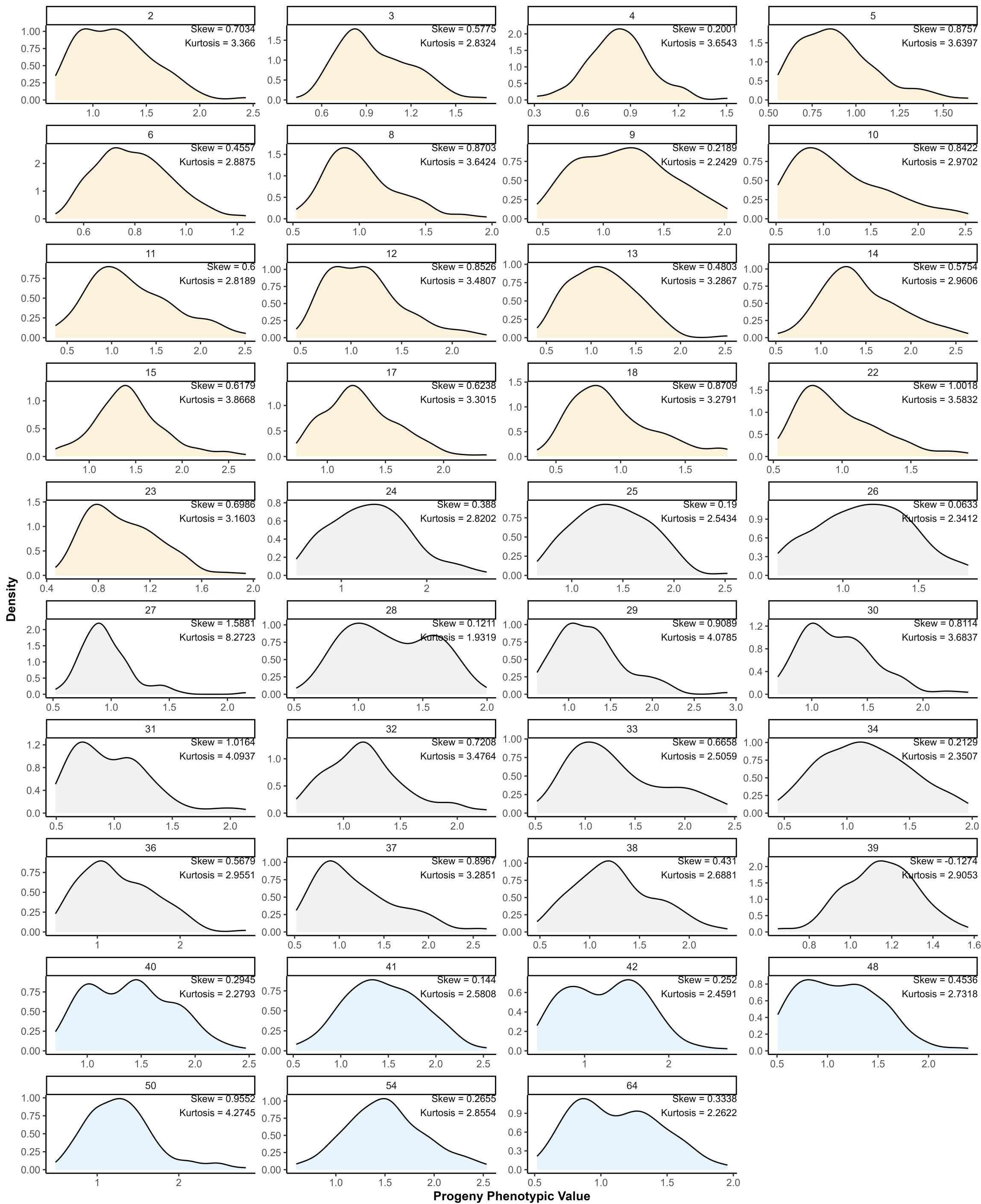

## Oil

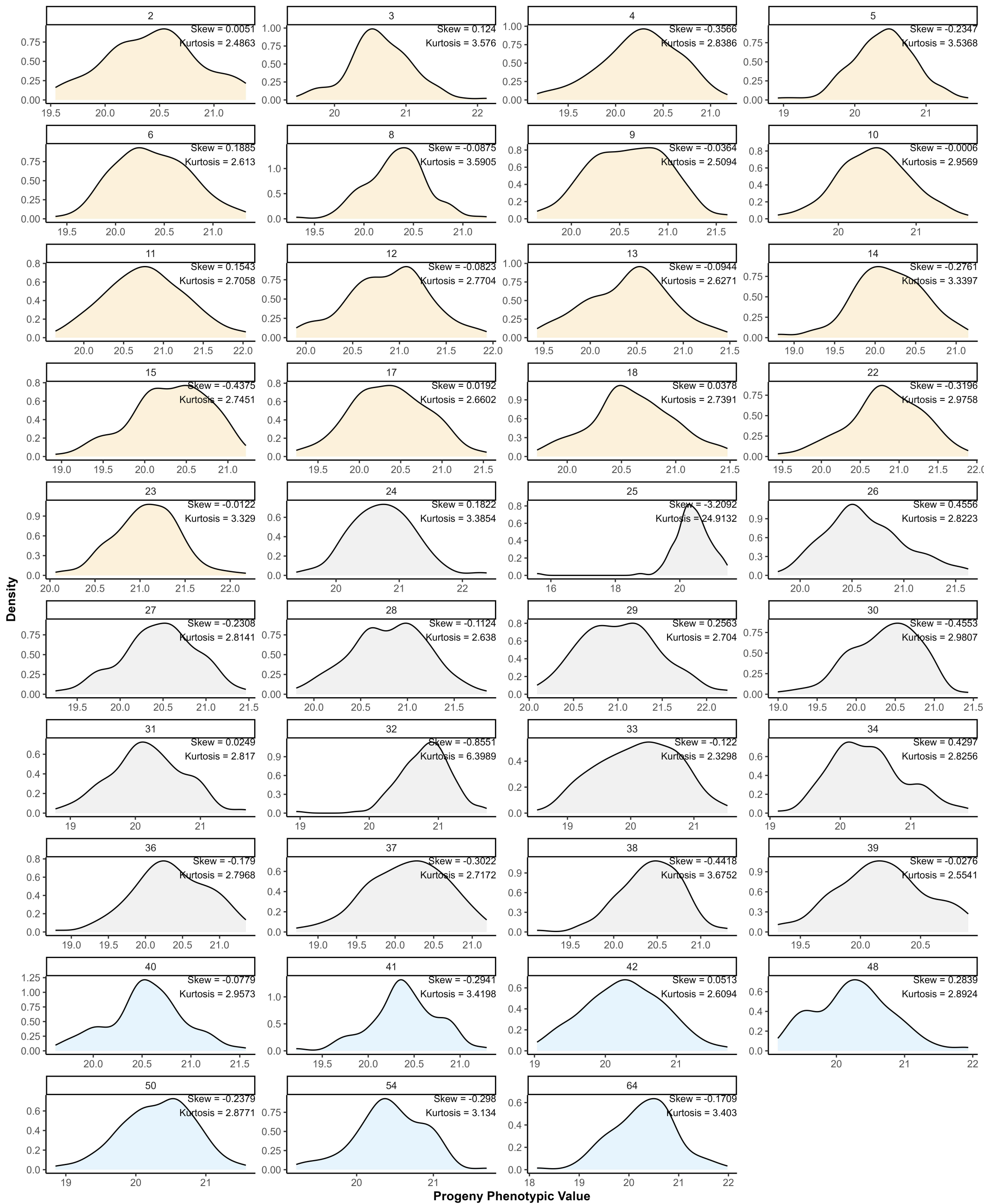

# Plant Height

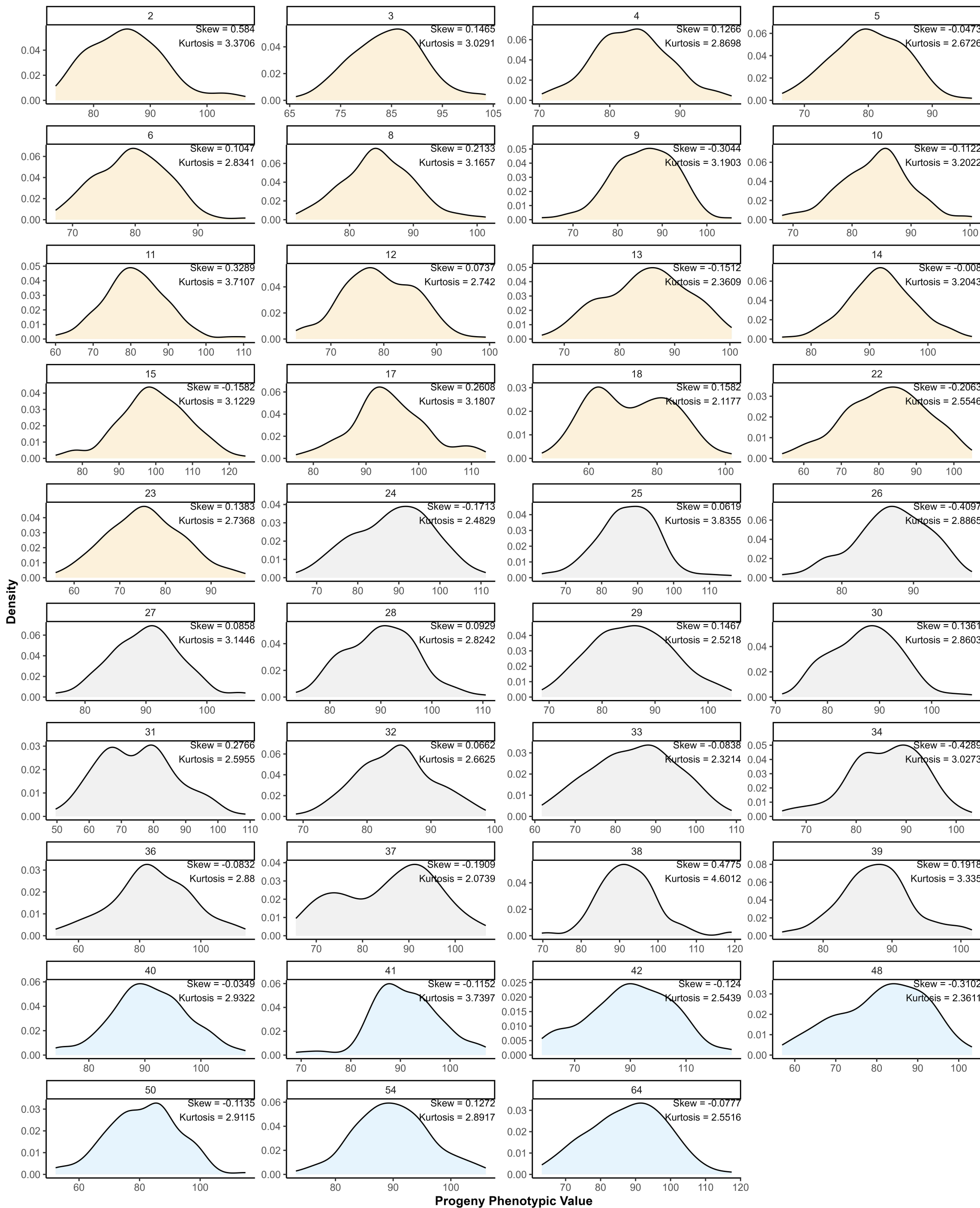

# Protein

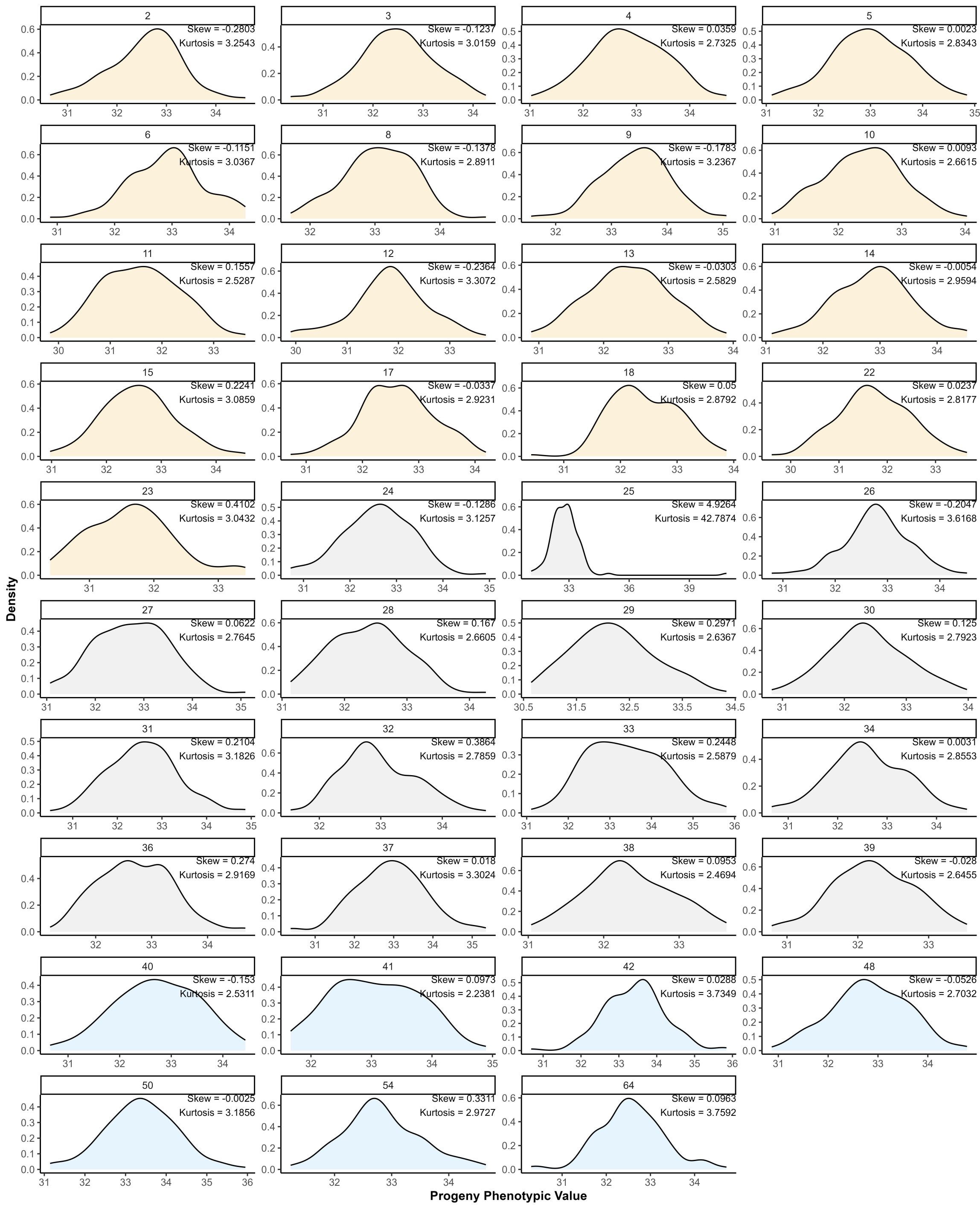

# Seed Size

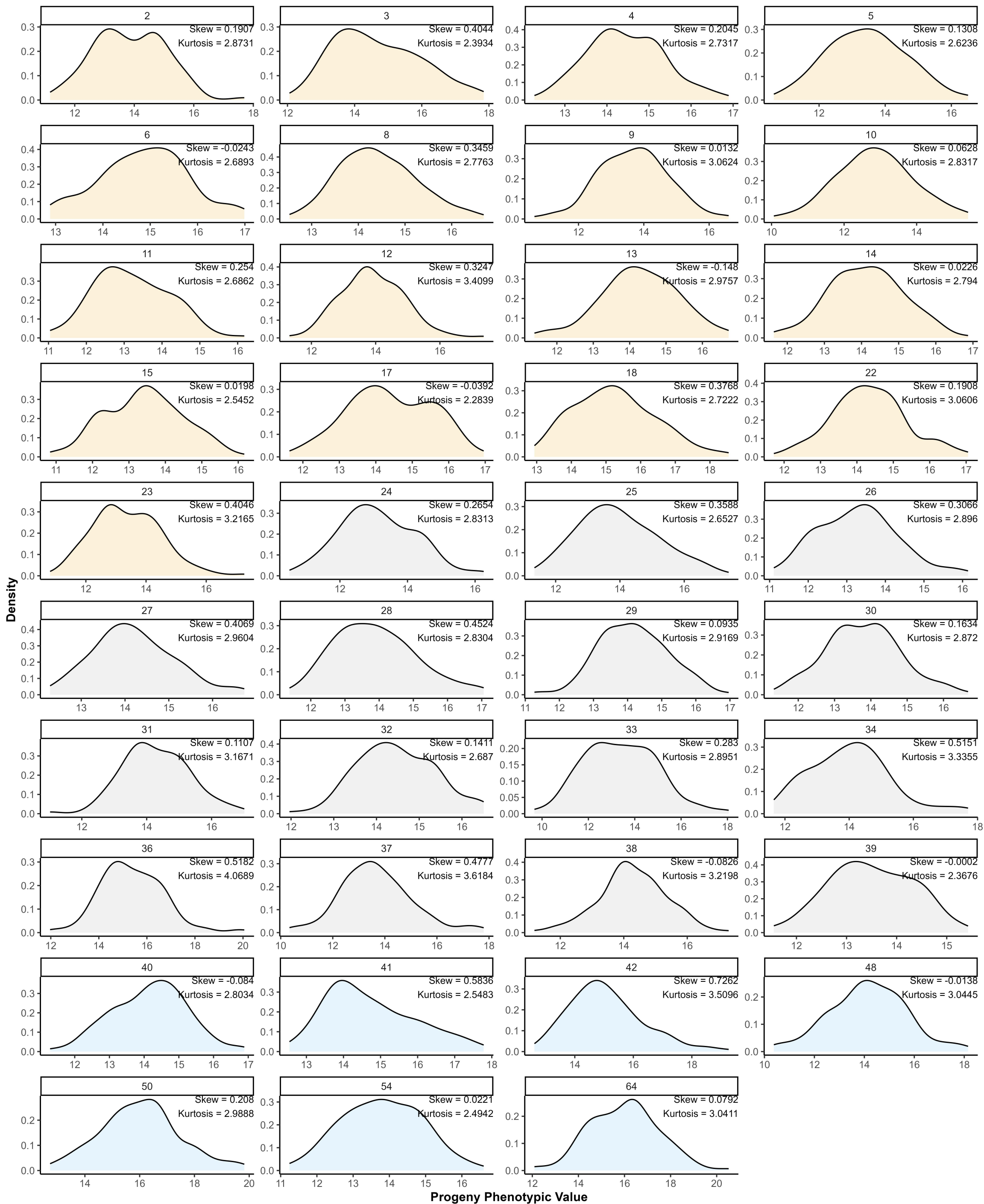

Supplement: Supplementary file 3 — Supplementary File 1 [file 41437_2024_703_MOESM3_ESM.pdf]
