# Supplementary material for: Genomic predictions of genetic variances and correlations among traits for breeding crosses in soybean
Source: Heredity (Edinb). 2024 Jul 12;133(3):173–85. doi: 10.1038/s41437-024-00703-3 (PMC11350137; doi:10.1038/s41437-024-00703-3)
Supplement: Supplementary file 4 — Supplementary table 1 [file 41437_2024_703_MOESM4_ESM.pdf]

Supplementary Table 1. Founders of the 40 SoyNAM families, along with their institution of origin, and group, according to Diers et al. (2018), number of RILs per family for which phenotypic data were available (N_pheno), and number of RILs per family for which molecular marker data were available (N_geno).

Entry	Family number	Origin	Group[a]	N_pheno	N_geno
IA3023	Hub-parent	Iowa State University	Common parent		
TN05-3027	2	University of Tennessee	EL	98	97
4J105-3-4	3	Purdue University	EL	137	135
5M20-2-5-2	4	Purdue University	EL	136	135
CL0J095-4-6	5	Purdue University	EL	139	139
CL0J173-6-8	6	Purdue University	EL	140	139
HS6-3976	8	Ohio State University	EL	138	137
Prohio	9	USDA-ARS, Wooster, OH	EL	137	137
LD00-3309	10	University of Illinois	EL	139	137
LD01-5907	11	University of Illinois	EL	124	121
LD02-4485	12	University of Illinois	EL	138	137
LD02-9050	13	University of Illinois	EL	137	137
Magellan	14	University of Missouri	EL	137	136
Maverick	15	University of Missouri	EL	139	138
S06-13640	17	University of Missouri	EL	135	133
NE3001	18	University of Nebraska	EL	136	136
Skylla	22	Michigan State University	EL	138	138
U03-100612	23	University of Nebraska	EL	140	140
LG03-2979	24	USDA-ARS, Urbana, IL	BX	140	136
LG03-3191	25	USDA-ARS, Urbana, IL	BX	122	118
LG04-4717	26	USDA-ARS, Urbana, IL	BX	108	108
LG05-4292	27	USDA-ARS, Urbana, IL	BX	132	130
LG05-4317	28	USDA-ARS, Urbana, IL	BX	139	112
LG05-4464	29	USDA-ARS, Urbana, IL	BX	138	137
LG05-4832	30	USDA-ARS, Urbana, IL	BX	138	138
LG90-2550	31	USDA-ARS, Urbana, IL	BX	127	125
LG92-1255	32	USDA-ARS, Urbana, IL	BX	138	137
LG94-1128	33	USDA-ARS, Urbana, IL	BX	134	133
LG94-1906	34	USDA-ARS, Urbana, IL	BX	133	129
LG97-7012	36	USDA-ARS, Urbana, IL	BX	137	134
LG98-1605	37	USDA-ARS, Urbana, IL	BX	130	111
LG00-3372	38	USDA-ARS, Urbana, IL	BX	132	131
LG04-6000	39	USDA-ARS, Urbana, IL	BX	134	133
PI 398881	40	South Korea	PI	137	131
PI 427136	41	South Korea	PI	99	96
PI 437169B	42	Russia	PI	133	108
PI 507681B	46	Uzbekistan	PI	0	0
PI 518751	48	Serbia	PI	139	138
PI 561370	50	China	PI	132	131
PI 404188A	54	China	PI	140	140
PI 574486	64	China	PI	124	118

[a]Founder group described as EL: elite, BX: breeding line with exotic ancestry, and PI: plant introduction.
